# Adult helpers increase the recruitment of closely related offspring in the cooperatively breeding rifleman

**DOI:** 10.1093/beheco/arw087

**Published:** 2016-06-15

**Authors:** Stephanie A.J. Preston, James V. Briskie, Ben J. Hatchwell

**Affiliations:** ^a^Department of Animal and Plant Sciences, University of Sheffield, Western Bank, Sheffield S10 2TN, UK and; ^b^School of Biological Sciences, University of Canterbury, Private Bag 4800, Christchurch 8140, New Zealand

**Keywords:** cooperative breeding, inclusive fitness, indirect fitness, kinship.

## Abstract

Indirect fitness benefits gained through kin-selected helping are widely invoked to explain the evolution of cooperative breeding behavior in birds. However, the impact of helpers on productivity of helped broods can be difficult to determine if the effects are confounded by territory quality or if the benefit of helpers is apparent only in the long term. In riflemen *Acanthisitta chloris*, helping and group membership are effectively decoupled as adult helpers are individuals that have dispersed from their natal territory and live independently from breeders in “kin neighborhoods.” Nevertheless, helpers direct their care toward close relatives, suggesting that helping provides indirect fitness benefits. The aim of this study was to examine the benefits of helpers to recipient offspring in the rifleman, investigating both short- and long-term effects. The total amount of food delivered to nestlings in helped broods was greater than that received by broods without helpers. This did not result in any short-term increase in nestling mass or nestling body condition nor was there any reduction in length of the nestling period at helped nests. However, helpers were associated with a significant increase in juvenile recruitment, with twice the proportion of fledglings surviving to the next breeding season from helped broods relative to unhelped broods. Thus, helpers gain indirect fitness by improving the survival of kin, and in contrast to a previous study of riflemen, we conclude that kin selection has played a key role in the evolution of cooperative breeding in this species.

## Lay Summary

In cooperative breeding systems, “helpers” assist in raising offspring that are not their own. In the rifleman, an endemic bird from New Zealand, we show that helpers increase the rate of food delivery to nestlings, which are often younger siblings, thereby increasing the likelihood that fledglings surviving to breeding age. Therefore, helpers gain indirect fitness benefits, and we conclude that kin selection has played a key role in the evolution of helping in this species.

## INTRODUCTION

In many cooperatively breeding species, helpers gain indirect fitness benefits by increasing the productivity of kin (Hamilton 1964). This can be achieved by enhancing the number or survival of offspring (Cockburn 1998), by improving the survival and hence the future reproduction of breeders (Crick 1992), or by enabling breeders to increase their number of reproductive attempts within a season (Russell and Rowley 1988). Kin selection is therefore considered to have played a significant role in the evolution of cooperative breeding (Brown 1987; Emlen 1991, 1995; Dickinson and Hatchwell 2004). However, in practice, demonstrating that helpers confer fitness benefits to recipient kin can be fraught with complications.

Disentangling the effects of help from the confounding effects of individual and territory quality can be difficult, particularly in those species where mature offspring typically remain on their natal territory to help. In such cases, the productivity of a breeding group or territory and the presence of helpers are intrinsically linked, making it difficult to distinguish cause from effect (Cockburn 1998; Dickinson and Hatchwell 2004; Cockburn et al. 2008; Wright and Russell 2008). In addition, although helpers sometimes improve the immediate survival or condition of recipient offspring (e.g., Waser et al. 1994; Hodge 2005), the fitness benefits are not always apparent in the short term. Instead, helpers may have downstream effects on recipient fitness that become evident only in the long term, such as increased survivorship to breeding age (Hatchwell et al. 2004), increased fecundity of helped offspring (Hodge 2005) or improved breeder survival rates (Meade et al. 2010). Helper effects may also be masked by subtle changes in breeder behavior and investment. For example, in superb fairy-wrens *Malurus cyaneus*, additional food provided by helpers does not increase offspring weight or survival (Dunn et al. 1995; Dunn and Cockburn 1996), but helped female breeders reduce their reproductive costs by laying smaller eggs, with the additional provisioning by helpers compensating for the initial reduced size of chicks (Russell et al. 2007). Furthermore, in some cases, the benefits of help may be apparent only under certain ecological conditions (Magrath 2001; Canario et al. 2004; Covas and du Plessis 2005; Koenig et al. 2011) that short-term studies may be unable to detect.

An important first step to identify the effects of helpers on recipients is to determine the causal mechanism by which helpers benefit breeders and/or offspring (Innes and Johnstone 1996; Magrath and Yezerinac 1997), primarily through understanding how breeders respond to the additional care provided by helpers (Hatchwell 1999; Heinsohn 2004). If breeders continue to provision nestlings at the same rate when helped, the care provided by helpers is additive to that of their own and may be expected to increase the number and/or quality of offspring produced, as in acorn woodpeckers *Melanerpes formicivorus* (Koenig and Mumme 1990; Mumme 1992) and apostlebirds *Struthidea cinerea* (Woxvold and Magrath 2005). In contrast, if breeders reduce their own parental effort when helped, the care provided by helpers is compensatory. For example, breeders may be the principal beneficiaries via reduced reproductive costs or “load-lightening,” resulting in increased survivorship and/or future fecundity (Crick 1992). Such fitness benefit gains by helped breeders have been noted in several cooperative breeders, including long-tailed tits *Aegithalos caudatus* (Hatchwell and Russell 1996; Meade et al. 2010), white-fronted bee-eaters *Merops bullockoides* (Emlen and Wrege 1991), and splendid fairy-wrens *Malurus splendens* (Russell and Rowley 1988). In addition, in some species, breeders only partially reduce their effort so that they receive some benefit from “load-lightening” while offspring also receive additive care from helpers (Hatchwell 1999; Kingma et al. 2010).

In this study, we examine whether adult helpers in the cooperatively breeding rifleman *Acanthisitta chloris* gain indirect fitness benefits from assisting kin. Rifleman pairs are sometimes assisted by sexually mature adult helpers and/or by juveniles from first broods who help to provision their siblings in second broods in the same season (Sherley 1990a; Preston, Briskie, et al. 2013). The demography of adult helpers is complex; both sexes help and they may be unpaired birds, failed breeders, or even successful breeders that simultaneously help at another nest (Preston, Briskie, et al. 2013). Importantly, these adult helpers are not delayed dispersers, but rather live independently from breeding pairs, joining them to help raise broods only in the nestling period (Sherley 1990a; Preston, Briskie, et al. 2013) where they often remain with helped offspring during an extended postfledging period of semi-independence. This presents us with an opportunity to examine the fitness benefits of helping in a species where helper presence and prior breeder success are not as intrinsically linked as in species exhibiting delayed dispersal.

An earlier study on riflemen assumed low kinship of adult helpers to helped broods and posited that helping by adults was driven by the direct fitness gain of raising future mates among the offspring that they helped (Sherley 1990a). However, the recent application of molecular genetic techniques to estimate relatedness showed that adult helpers were usually grown offspring of the breeders that visited their parents’ territory to help provision younger siblings, effectively ruling out the “future mates” hypothesis, and instead suggesting the potential for indirect fitness to be accrued by helpers (Preston, Briskie, et al. 2013).

Here, we established the mechanism by which adult rifleman helpers might benefit recipients by examining whether helpers increase the overall rate of nestling provisioning or if breeders take advantage of help to reduce their own provisioning rates. We also examined whether adult helpers have a measurable short-term effect on nestling mass or condition, which could influence survival in the short term, to fledging age, or in the long term, to breeding age (e.g., Hatchwell et al. 2004; Brouwer et al. 2012). We investigated whether the nestling period was shorter when helpers were present, as reported in some other cooperative breeders (e.g., Ambrose and Davies 1989; Strahl and Schmitz 1990); this may confer benefits to breeders by reducing the costs of feeding completely dependent nestlings or by increasing the probability of producing a second brood in the same season. Finally, to determine whether adult rifleman helpers are associated with an increase in the productivity of helped breeders, we examined whether broods with helpers were larger than those without helpers, and also the effect of helpers on the local survival of offspring to reproductive age (i.e., recruitment rate).

## METHODS

### Study system

We studied a population of 15–30 breeding groups of riflemen for 3 breeding seasons (September–January) between 2008 and 2011 at Kowhai Bush, in Kaikoura, New Zealand (42°22′34″S, 173°36′58″E). Kowhai Bush is a 240 ha block of native New Zealand forest composed of seral kanuka *Kunzea ericoides* forest with little understorey. The rifleman is an endemic insectivorous passerine of New Zealand with an adult mass of 5–7g. They form long-term pair bonds that endure through the nonbreeding season. Riflemen occupy small home-ranges year-round that overlap little with other pairs during the breeding season, even though they rarely show aggressive territorial behavior (Hunt and McLean 1993). The average lifespan is 2.2 years (Sherley 1994), but some individuals in this study lived for at least 4 years. Riflemen breed from September to January and can rear up to 2 broods per season. Eggs are laid at 2-day intervals with first brood clutch sizes typically 3–5 eggs and second brood clutches of 2–4 eggs (Higgins et al. 2001). Eggs hatch synchronously after 19–21 days of biparental incubation. Both parents provision nestlings with small adult and larval invertebrates, such as moths, spiders, and crickets that are delivered singly to the nest. Provisioning rates typically peak when nestlings are 15–18 days old, when on average males visit about 16 times per hour and females 10 times per hour (Preston, Briskie, et al. 2013).

Helpers may start assisting pairs at any time during the typical 24-day nestling period or during the 4–5 weeks postfledging period of dependence. Once a helper appears, they usually continue provisioning until fledging, although not necessarily consistently. Helpers can be of either sex and may be adult (fledged in a previous breeding season and observed at both first and second broods) or juvenile (fledged in the same breeding season and observed caring for second broods). Helpers generally provision broods less frequently than breeders, and there is no difference between the sexes (Preston, Briskie, et al. 2013). Juvenile helpers provision at significantly lower rates than adult helpers (Preston, Briskie, et al. 2013) so the 2 age classes are not equivalent.

### Data collection

Adult riflemen were captured in mist-nets and banded with a unique combination of colored rings plus a metal ring so that individuals could be identified. Adults were caught prior to egg laying or posthatching to minimize the risk of nest abandonment. Most nests were in one of the 230 nest boxes available at the field site although a few were located in natural cavities (8 of 81 nests containing eggs). All breeding attempts were closely monitored by checking occupied nest boxes at regular intervals from the first signs of nest building until broods fledged or the nest failed; nest checks were made daily during egg laying and around hatching and fledging.

In this study, we consider only the effect of adult helpers attending first broods. Just one of the 11 second broods that we monitored did not have a juvenile helper so the effect of juvenile helpers on recipients could not be analyzed. Eleven of 58 (19%) first broods that fledged young in this study were attended by 1–2 adult helpers (mean = 1.4). Helpers were observed assisting only a single pair within a season, although 5 of 11 adult helpers also simultaneously provisioned their own offspring (fledglings in 4 out of 5 cases).

We used digital camcorders to record provisioning rates of carers during a 1-h observation period between 0700 and 1700 every third day from day 3 (day of hatching = day 0) until nestlings fledged (up to day 24) or the nest failed. To minimize disturbance, cameras were placed in a sheltered place at least 10 m away from the nest, and a 10-min habituation period was implemented before data collection commenced.

Following the nest observation period on day 15, all nestlings were color banded, blood sampled, weighed (to 0.1g), and tarsus length measured (to 0.1mm). In addition, we weighed nestlings in accessible nests after every provisioning watch from day 3 to day 18, after which the risk of inducing premature fledging rises (Sherley 1993). Riflemen nestlings could be sexed in the hand at pin break, but the sex of all individuals was also confirmed by genotyping using the Z043B microsatellite marker (Dawson DA et al., unpublished data; Preston, Briskie, et al. 2013; Preston, Dawson, et al. 2013). Brood size was recorded at least every third day. To determine fledge date, we checked nests each afternoon (most directly observed fledging events occurred 0900–1300h) from day 20 onwards. Chick mortality and/or nest failure were recorded and the cause of death noted where possible,

To assess individual survivorship between years, we conducted thorough searches of the field site at the start of each breeding season and regularly throughout the rest of the season. Unlike most other bird species, this population of riflemen exhibits limited dispersal in both sexes and immigration and emigration events are rare (Sherley, 1993; Preston SAJ, unpublished data). Thus, it could be assumed with reasonable confidence that failure to resight a bird was a result of mortality rather than dispersal.

### Statistical analyses

#### Do helpers affect parental provisioning rates?

To assess whether parents adjusted their provisioning rates when helped we fitted a linear mixed-effects model with normal error structure and identity link function to data from 287 observation hours of 68 parents at 51 different nests. We used a square-root transformation of individual parental provisioning rate (number of visits per hour) as the response variable, and fitted helper presence as an explanatory binary variable “helped” (1 if nestlings were provisioned by a nonparent, and 0 if only parents provisioned within an observation period). Seven of the 11 helped nests had only a single helper, and of the remaining 4 nests only one had 2 helpers at the same time in more than 2 observation periods; therefore, we did not use helper number as an explanatory variable. We included the following explanatory terms as fixed effects in the maximal model: nestling age, nestling age^2^, brood size, date, time, temperature, and parent sex. Nestling age was the number of days from hatching (day 0), and we included the term nestling age^2^ because parental provisioning in riflemen shows a curvilinear response to nestling age (Preston, Briskie, et al. 2013). Brood size was the number of nestlings on the day of observation. Date was the number of days after 1 September each year, and time was recorded as the hour the observation period started. Ambient temperature at the start of each observation period was extracted from records of the Kaikoura Weather Station (42°25S, 173°42E). We included the sex of the parent as a fixed effect because male riflemen provision at higher rates than females (Sherley 1990b; Preston, Briskie, et al. 2013). We also fitted an interaction between “helped” and “parent sex” because male and female parents may respond differently to provisioning by helpers (Hatchwell 1999). In addition, we fitted interactions between “helped” and brood size, and “helped” and nestling age terms (linear and quadratic) because the parental response to help may depend on the demands of the brood (e.g., Meade et al. 2010). We also included an interaction between “parent sex” and nestling age terms to allow for a difference in provisioning response to older nestlings between male and female breeders. We fitted parent identity and nest identity as random factors to control for the nonindependence of data taken from repeated observations of the same individual and nest. We included parent identity nested within nest identity as a random effect to account for parents with more than one nest during the study period.

All statistical analyses in this study were performed in R v 2.13.1 (R Development Core Team 2008). We fitted this model and all other linear mixed models described in this study using the package lme4 (Bates et al. 2008). To obtain the minimum adequate model, we fitted maximal models using a maximum-likelihood approach and removed terms by backward stepwise deletion based on the Akaike information criterion (AIC). Terms with the lowest χ^2^ values were removed if it resulted in a model with a significantly lower AIC value. Once the final model was obtained, we used a forward-checking method to add deleted terms back into the model to determine the level of nonsignificance and to check terms had not been dropped incorrectly. The final model was refitted using a restricted maximum-likelihood approach to gain estimates and probabilities for significant terms.

#### Do helpers increase the rate of provisioning of nestlings?

To determine whether helpers increased the total rate of food delivery to nestlings we fitted a linear mixed-effects model with normal error structure and identity link function to data from 280 observations from 51 nests. We used a square-root transformation of total provisioning rate (number of visits made by all carers combined per hour) as the response variable, and fitted the binary variable “helped” as an explanatory term as in the previous model. We fitted brood size, nestling age, nestling age^2^ as fixed effects and controlled for date, time of observation and temperature in the maximal model. We included interaction terms between “helped” and nestling age terms, and between “helped” and brood size in the maximal model. The model also contained nest identity as a random variable to account for repeated observations of the same nest. Eight of the 39 pairs had nests in multiple years so we included pair identity as a random factor also.

#### Do helpers increase nestling growth?

**Figure 1:**
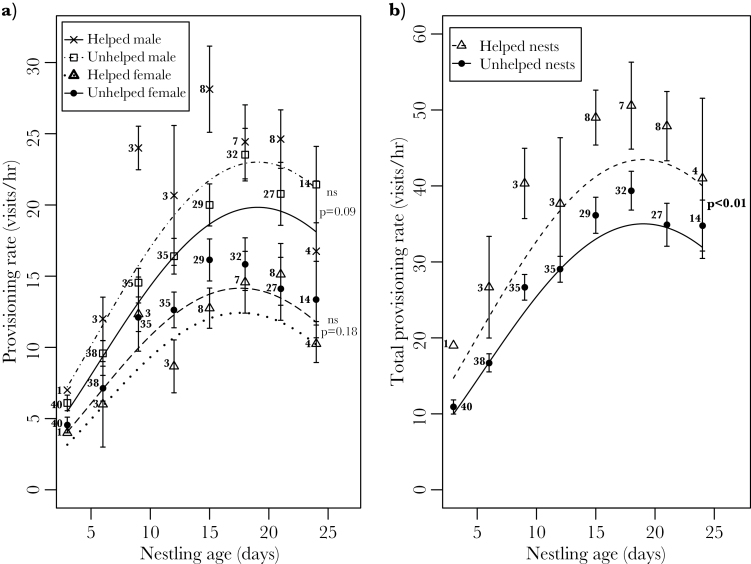
The effect of provisioning helpers on (a) parental provisioning rates and (b) total provisioning rates throughout the nestling period. Points show real data (means ± SE). Lines show predicted values of provisioning rates from the minimum adequate models (Table 1 and 2) with all other explanatory variables set to their median values. Predicted values are back-transformed from estimates in the models. Numbers represent sample sizes.

We assessed the effect of helpers on the mass and body condition of male and female nestlings separately because rifleman nestlings are sexually dimorphic (mean mass at day 15: males = 7.64g ± 0.052 standard error [SE], females = 8.75g ± 0.053; Sherley 1993). For each analysis, we fitted a linear mixed model with normal error structure and link function to data from 78 female nestlings from 38 broods, and then to data from 61 male nestlings from 36 broods. For the response variable “nestling mass,” we used the mass of chicks measured on day 15 of the nestling period, which was then zero centered around the mean. The growth of rifleman nestlings plateaus at day 15 and is a good indication of relative size at fledging (Figure 1). For the response variable “nestling body condition,” we calculated the residuals from a regression of body mass at day 15 of the nestling period against tarsus length (Brown 1996). We fitted “helped” as a fixed effect, which in these analyses was a binary variable, where values were set to 1 only if nestlings were provisioned by a nonparent before day 15 and to 0 if provisioned by parents alone in the first 15 days. We included brood size (number of nestlings at day 15), sex ratio (proportion of male nestlings within a brood), and hatch date (days after 1 September) as fixed effects in the maximal model. We included sex ratio because it affects nestling mass and condition in some species (e.g., Nam et al. 2011). We also tested for an interaction between “helped” and “brood size.” To account for nonindependence of data from nestlings within the same brood, we included nest identity as a random effect. We did not include pair identity as a random factor as few pairs raised more than one brood over the course of the study.

To test the effect of helpers on the length of the nestling period, we performed an analysis of covariance using residuals from the mean fledging day (day 0 = day of hatching) as the response variable. We fitted the explanatory binary variable “helped” (1 if a helper had provisioned at the nest at any time in the nestling period; 0 if only parents provisioned the nest). We fitted brood size (number of chicks on day 15 of the nestling period), hatch date, and year as covariates and included an interaction between “helped” and “brood size” in the maximum model. In order to obtain the minimum adequate model, we sequentially removed the least significant terms from the model and compared models using *F* values generated by analysis of variance tests.

#### Do helpers improve offspring survival?

Comparison of breeding success with and without helpers is complicated in species where helpers may appear at any time during the nestling period. A simple comparison of the success/failure rates of helped and unhelped nests is meaningless because nests that fail early do not have chance to acquire helpers, whereas those that survive are more likely to be helped, resulting in a false-positive effect of helpers on breeder success. As helpers are not present prior to the nestling period, they are also unlikely to influence the clutch and hence initial brood sizes. On the other hand, we were able to compare the brood size at hatching and fledging in nests with and without helpers to investigate whether helpers increased the number of fledglings produced. Total nest failures were due to either depredation from invasive mammals or early abandonment of small broods. As these nests did not survive long enough to accrue helpers (and as helpers were unlikely to be able to influence either event), we excluded them and compared only 42 nests that survived until fledging.

To examine the effect of helpers on the probability of recruitment, we used a linear mixed-effects model fitted to data from 104 fledglings from 32 broods. We defined the response variable “survival” as the survival of a fledgling to the next breeding season (resighted from 20 September onwards in the subsequent year) and fitted this as a binary variable with a logit link function. Note that for the 2008–2009 cohort, all recruits were observed in the year following fledging (2009–2010) rather than a later year (2010–2011), so we are confident that we did not overlook local recruits in their first year. We defined the explanatory binary variable “helped” in 2 different ways. In the first model, values were set to 1 if fledglings were provisioned by a helper at any stage of the nestling period and to 0 if provisioned only by parents. In the second model, values were set to 1 only if helpers started provisioning on, or prior to, day 15 of the nestling period and 0 if broods were never helped or if helpers turned up later than day 15 (i.e., the median day of helper appearance at first brood nests). In this model, 2 nests where helpers were first observed on day 21, that is, shortly before fledging, were moved from the “helped” to “not helped” category. In both cases, the helpers were feeding their own offspring simultaneously and provided very little care for the helped brood. For each analysis, we included brood size (number of nestlings at day 15), nestling sex, hatch date, and year as fixed effects in the maximal models for both analyses. We also tested for an interaction between “helped” and “brood size” and between “helped” and “nestling sex.” In some studies the effect of brood size on offspring mortality is sex-dependent (DeKogel 1997), so additionally we tested for an interaction between “nestling sex” and “brood size.” We fitted nest identity as a random factor. Only 4 of 28 pairs fledged broods in both years so we did not include pair identity as a random variable.

## RESULTS

### Do helpers affect parental provisioning rates?

When the effect of helpers on parental provisioning rates was analyzed with both parents in the same model, there was a significant interaction between “helped” and parental sex (Table 1, Figure 1a), indicating that females decreased their provisioning rate when helped whereas males increased their provisioning rate when helped (Figure 1a). However, when the sexes were analyzed separately, there was no significant difference between provisioning rates of parents at helped and unhelped nests (males: χ^2^ = 2.96, degrees of freedom [df] = 1, *P* = 0.09; females: χ^2^ = 1.78, df = 1, *P* = 0.18), suggesting that the significant interaction between sex and “helped” was due to the different directions of marginal change in provisioning rates by male and female breeders. Therefore, the evidence that the presence of a helper lightened the load of rifleman parents was equivocal, but suggestive of no strong effect.

**Table 1:** Factors affecting parental provisioning rates

	Parental provisioning rate √visits/hour			
Random effects	Variance			
Parent identity	0.100			
Nest	0.034			
Parent identity | nest	<0.001			
Residual	0.532			

Fixed effects	Estimate ± SE	df	χ^2^	*P*
Intercept	0.398±0.306			
Nestling age	0.288±0.022	1		
Nestling age^2^	−0.008±0.001	1	92.86	**<0.001**
Brood size	0.339±0.047	1	40.09	**<0.001**
Sex (M)	0.835±0.292	1		
Helped (unhelped)	0.235±0.181	1		
Time	−0.042±0.013	1	10.26	**0.001**
Nestling age | sex (M)	0.023±0.009	1	5.78	**0.02**
Helped | sex (unhelped × M)	−0.577±0.248	1	5.46	**0.02**
Temperature	−0.002±0.005	1	0.27	0.60
Date	−0.001±0.003	1	0.23	0.64

Estimates calculated from a generalized linear mixed model with normal error structure. The intercept refers to a baseline of an unhelped female breeder with estimates calculated from the named factorial fixed effects where relevant. Significant terms (*P* < 0.05) were retained in the minimum adequate model. Estimates were calculated by refitting the model using a REML approach. *P* values and χ^2^ values were estimated by removing variables and model comparisons using a ML approach. For nonsignificant terms, estimates, *P* values, and χ^2^ values were calculated by adding each to the minimum adequate model individually. Interactions detailed in the methods were included in the maximal model, but only significant interactions in the final model are reported. Significant values (*P* < 0.05) are reported in bold. REML, restricted maximum-likelihood.

### Do helpers increase the amount of food delivered to nestlings?

The total provisioning rate at helped nests was significantly higher than at nests provisioned solely by parents (Table 2, Figure 1b), with offspring at helped nests receiving more food than those at unhelped nests throughout the nestling period.

**Table 2:** Factors affecting total provisioning rates to nests (food delivered by all carers)

	Total provisioning rate √visits/hour			
Random effects	Variance			
Nest	0.112			
Pair identity	<0.001			
Residual	0.671			

Fixed effects	Estimate ± SE	df	χ^2^	*P*
Intercept	−1.817±0.401			
Nestling age	0.409±0.034	1		
Nestling age^2^	−0.011±0.001	1	62.38	**<0.001**
Brood size	0.532±0.067	1	46.82	**<0.001**
Helped (unhelped)	−0.67±0.188	1	12.38	**<0.001**
Time	−0.071±0.021	1	11.62	**<0.001**
Temperature	−0.003±0.008	1	0.16	0.69
Date	−0.002±0.004	1	0.44	0.51

Estimates from a generalized linear mixed model with normal error structure. The intercept refers to a baseline of a “helped” nest. Significant terms (*P <* 0.05) were retained in the minimum adequate model and are reported in bold. Estimates were calculated by refitting the model using a REML approach. *P* values and χ^2^ values were estimated by term deletion and model comparison using a ML approach. For nonsignificant terms, estimates, *P* values, and χ^2^ values were calculated by adding each to the minimum adequate model individually. Only significant interactions are reported. REML, restricted maximum-likelihood.

### Do helpers increase nestling growth?

Despite providing extra food to offspring, helpers had no significant effect on the mass or condition of nestlings (Table 3). Only hatch date had a significant, but small, effect on female nestling mass and condition, with earlier nestlings weighing more and being in better condition than later nestlings (Table 3). Furthermore, the length of the nestling period (mean ± SE = 24.38±0.19 days) was not related to the presence or absence of helpers (Table 4). Brood size was the only factor close to significance, suggesting that larger broods may fledge slightly earlier than smaller broods (Table 4).

**Table 3:** Factors affecting (a) nestling mass and (b) nestling condition

(a)								
	Female nestling mass (g)				Male nestling mass (g)			
Random effects	Variance				Variance			
Nest	0.043				0.044			
Residual	0.177				0.119			
Fixed effects	Estimate ± SE	df	χ^2^	*P*	Estimate ± SE	df	χ^2^	*P*
Helped	−0.220±0.151	1	2.21	0.46	0.056±0.129	1	0.21	0.65
Brood size	0.016±0.079	1	0.04	0.84	−0.018±0.068	1	0.08	0.78
Sex ratio	−0.267±0.274	1	1.00	0.32	0.205±0.253	1	0.67	0.41
Date	−0.009±0.004	1	5.66	**<0.02**	−0.005±0.003	1	2.03	0.15
(b)								
	Female nestling condition				Male nestling condition			
Random effects	Variance				Variance			
Nest	0.040				0.044			
Residual	0.166				0.118			

Fixed effects	Estimate ± SE	df	χ^2^	*P*	Estimate ± SE	df	χ^2^	*P*
Helped	−0.238±0.145	1	2.78	0.10	0.074±0.127	1	0.37	0.54
Brood size	0.027±0.077	1	0.13	0.72	−0.018±0.063	1	0.01	0.97
Sex ratio	−0.304±0.265	1	1.39	0.23	0.210±0.251	1	0.73	0.40
Date	−0.009±0.003	1	6.69	**<0.01**	−0.005±0.003	1	2.23	0.14

Estimates calculated using a general linear mixed-effects model (normal error structure and identity link). Significant terms (*P <* 0.05) were retained in the final model and are reported in bold. Term estimates were calculated by refitting the model using a REML approach. *P* values and χ^2^ values were estimated by term deletion and model comparison using a ML approach. For nonsignificant terms, estimates, *P* values and χ^2^ values were calculated by term addition and model comparison. Only significant interactions are reported. REML, restricted maximum-likelihood.

**Table 4:** Factors affecting the length of the nestling period in riflemen as calculated from an analysis of covariance

		Nestling period (days)		
Effects	Estimate ± SE	*F*	df	*P*
Helped	0.11±1.51	0.24	1,41	0.70
Brood size	−0.40±0.24	3.94	1,41	0.054
Date	<−0.01±0.11	0.11	1,41	0.74
Helped | brood size	0.04±0.43	2.10	2,41	0.14

### Do helpers improve offspring survival?

Of the 56 broods monitored until fledging, 14 (25%) suffered total nest failure. Four suffered total depredation (mean ± SE nestling age = 8.5±2.9 days). Six small broods were spontaneously abandoned (mean ± SE brood size = 1.5±0.3 chicks, mean ± SE nestling age = 6.0±1.1 days). Four broods suffered partial depredations and subsequent abandonment of surviving nestlings (mean ± SE nestling age = 9.0±1.4 days). Nestling mortality rates from other causes were low. Five nests (9%) suffered partial brood loss from chicks falling out of the nest, starvation, or from unknown causes (mean ± SE number of nestlings lost per nest = 1.8±0.4, mean ± SE nestling age at death = 9.5±1.5 days).

In nests that survived to fledging the brood size at hatching was not significantly different between nests that subsequently gained helpers (mean ± SE = 3.8±0.4 nestlings, *n* = 11) and those that did not (mean ± SE = 3.5±0.2 nestlings, *n* = 31; Wilcoxon test, *W* = 125, *P* = 0.17). Likewise, there was no significant difference in the number of fledglings produced from helped nests (mean ± SE = 3.5±0.3 fledglings, *n* = 11) and nests without helpers (mean ± SE = 3.3±0.2 fledglings, *n* = 31; Wilcoxon test, *W* = 147, *P* = 0.48).

**Figure 2:**
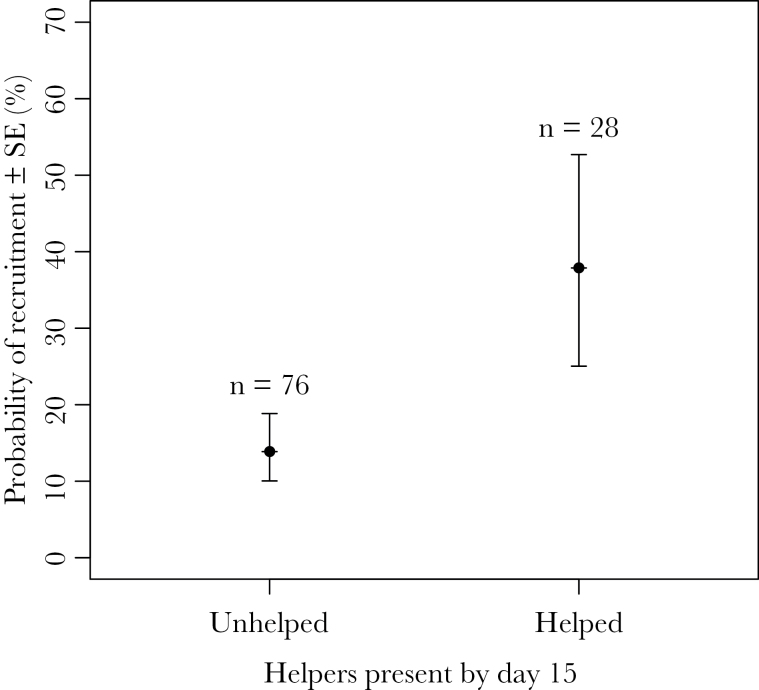
Predicted probability of recruitment of fledglings from “unhelped” and “helped” nests. “Helped” nests are defined as those with helpers appearing from day 15 or before of the nestling period. Predicted values (± SE) from a linear mixed-effects model (Table 5). Numbers indicate sample sizes.

The overall proportion of fledged offspring that recruited into the population from the first 2 years’ cohorts was 22.2% (*n* = 104). Fledged juveniles from helped broods were more likely to recruit the following year, but this was significant only when helpers had started helping by day 15 of the nestling period (Table 5, Figure 2), that is, when the 2 broods, where a helper appeared on day 21, were excluded. The predicted recruitment probabilities of 37.8% for helped fledglings and 13.9% for unhelped fledglings (Figure 2) are close to the observed recruitment rates of 39% (*n* = 28) and 15.7% (*n* = 76) for helped and unhelped fledglings, respectively. Offspring recruitment was not significantly related to any other factor, including sex.

**Table 5:** Factors affecting recruitment rate of fledged offspring where the effect “helped” in the GLMM was defined by (a) if broods were helped at point during the nestling period and (b) if nests were attended by helpers by day 15 of the nestling period

Recruitment rate of fledged offspring								
	(a) Helpers present at any point?				(b) Helpers present by day 15?			
Random effects	Variance ± SD				Variance ± SD			
Nest	1.058±1.028				0.450±0.671			

Fixed effects	Estimate ± SE	df	χ^2^	*P*	Estimate ± SE	df	χ^2^	*P*
Helped	1.071±0.601	1	2.91	0.09	1.375 **±** 0.529	1	4.75	**0.03**
Brood size	−0.503±0.392	1	1.44	0.23	−0.650±0.364	1	2.93	0.09
Sex (M)	−0.186±0.532	1	0.12	0.73	−0.369±0.529	1	0.48	0.49
Date	−0.008±0.019	1	0.17	0.68	−0.005±0.163	1	0.08	0.77

Recruitment (survival to the next breeding season) was fitted as a binary variable with a logit link function. Significant terms (P < 0.05) were retained in the final models and are reported in bold. Term estimates were calculated by refitting the models using a REML approach. *P* values and χ^2^ values were estimated by term deletion and model comparison using a ML approach. For nonsignificant terms, estimates, *P* values, and χ^2^ values were calculated by term addition and model comparisons. GLMM, generalized linear mixed model; SD, standard deviation; REML, restricted maximum-likelihood.

## DISCUSSION

To determine whether helpers gain indirect fitness benefits in cooperatively breeding species, it must be shown that helping is directed toward relatives and that helpers have a positive effect on the productivity of these kin. A previous study revealed that most riflemen helpers are closely related to the recipients of their help (Preston, Briskie, et al. 2013). In this study, we show that care by rifleman helpers results in additional food being delivered to nestlings and that helpers are associated with the long-term benefit of a substantial increase in the recruitment of fledglings. Together these results indicate that indirect fitness benefits gained by helpers are an important factor in the evolution of cooperative breeding in riflemen.

Overall provisioning by helpers was additional to that by parents, and consequently, nestlings in helped broods received more food than their unhelped counterparts (Figure 1b, Table 2). This effect was evident in broods of all sizes, and throughout the nestling period. Our evidence for load-lightening, on the other hand, was equivocal. Although the analysis that considered male and female responses together suggested a divergent response of male and female breeders to helpers (Figure 1a, Table 1), these patterns disappeared when male and female breeder parental provisioning were analyzed separately. Female-biased load-lightening is rare among cooperative breeders (Hatchwell 1999), although it has been reported in pygmy nuthatches *Sitta pygmaea* (Sydeman 1989). Similarly unusual is the response of male breeders to work harder when assisted by a helper, an effect that has only been documented previously in the Iberian magpie *Cyanopica cooki* (Valencia et al. 2006).

Optimal parental investment is determined by a trade-off between the fitness benefits of investing in a current brood and the costs to residual reproductive success (Williams 1966; Trivers 1972). Additive care by helpers is predicted to occur in species with frequent nestling starvation, where the benefits of additional help to current reproductive success might be maximized, whereas load-lightening is predicted to occur where nestling starvation is rare and parents might benefit more by conserving resources for future reproductive attempts (Hatchwell 1999). Many single species studies support this prediction (e.g., Emlen and Wrege 1991; Heinsohn 1992; Luck 2002; Woxvold and Magrath 2005) although there are some exceptions (e.g., Langen and Vehrencamp 1999; Legge 2000) where load-lightening occurs despite high nestling starvation rates. Riflemen may also be an exception, although in this case care by helpers is at least partially additive even though starvation is not a major cause of nestling loss (starvation occurred in only 9% of nests, causing an average brood reduction of 28% in those nests). It is possible that the rifleman’s short life span (few survive beyond 4 years) limits opportunities for future reproduction. However, riflemen often raise a second brood within a year, so breeders should take the opportunity to reduce first brood costs to increase their chances of renesting. An interesting direction for future research would be to examine whether parental investment rules in response to help differ according to the likelihood of raising a second brood.

Our results show that nestlings, rather than parents, are the main beneficiaries of helper care. However, despite finding that helped nestlings receive more food than unhelped nestlings, we found no discernible effect of help on nestling mass or condition. Although it appears intuitive that increased delivery of food should result in heavier offspring, as found in some species (e.g., Hatchwell et al. 2004; Covas and du Plessis 2005; Hodge 2005; Lloyd et al. 2009), this finding is not universal. One explanation argued in the case of white-browed scrubwrens *Sericornis frontalis* (Magrath and Yezerinac 1997) is that delivery of extra food to nestlings does not increase growth either because there are physical limitations to size or because it would result in a supraoptimal weight (Gosler et al. 1995). It was also suggested that higher feeding rates may lead to a decreased length of the nestling period, although no such effect was found in the white-browed scrubwrens (Magrath and Yezerinac 1997) or in this study.

The additional food provided by riflemen helpers does not result in an increase of absolute mass of offspring, but it could positively influence the rate of growth and development, ensuring that nestlings reach a critical mass or stage of development sooner, for example, allowing them to thermoregulate at an earlier age. We were unable to investigate this possibility here because many nestlings were weighed only at day 15 of the nestling period, where nestling mass typically asymptotes (Figure 1).

Depredation and spontaneous abandonment of small broods were the major causes of nestling mortality, both of which are unlikely to be influenced by the presence of helpers. Nestling mortality rates from other causes were notably low. Unless nests suffered the catastrophic failures detailed above, the vast majority of chicks survived to fledging so we found no difference between the number of fledglings produced at helped and unhelped nests. Our key finding was that fledglings from helped nests had a substantially higher probability of recruitment (39%) than those from unhelped nests (16%). This association was significant only when 2 nests with late-arriving helpers were excluded from the helped category. In both cases, these helpers were breeders with their own fledglings to care for and help was limited to the final few days of the nestling period and did not extend into the postfledging period. A positive impact of helpers on offspring recruitment has been reported in several species (e.g., Emlen and Wrege 1989; Hatchwell et al. 2004; Ridley 2007), but the mechanisms underlying this benefit are unclear. In some studies, helpers had a positive impact on offspring mass and this is likely to be correlated with future survival (Garnett 1981; Magrath 1991; Hodge 2005). Few avian studies, however, have examined the postfledging care of individuals by helpers, despite the fact that this period of care can often be longer that the nestling period (for exceptions, see Heinsohn 1991; Langen 1996; Ridley 2007). Ridley (2007) showed that Arabian babbler *Turdoides squamiceps* helpers improved both postfledging weight gain and the development of juveniles’ foraging skills. Rifleman helpers often provision offspring during the postfledging period when juveniles are attaining independence (Sherley 1990a). However, nothing is known about how additional care at this time benefits offspring. At peak mass rifleman nestlings are up to 20–35% heavier than adults, but juveniles lose most of this extra weight during the first few weeks after fledging (Preston SAJ, personal observation); helpers may mitigate this weight loss, as found in Arabian babblers (Ridley 2007). Helpers may also protect relatively immobile and vulnerable fledglings against predators through mobbing or alarm calling. To determine how riflemen helpers confer a survival advantage to post-fledged offspring, we suggest future attention should be directed to this little studied period of cooperative care.

The association between future recruitment and help is important because average relatedness between riflemen helpers and recipient offspring is high (mean *r* = 0.43, Preston, Briskie, et al. 2013), so helpers stand to gain substantial indirect benefits. In species where offspring delay dispersal to become helpers or where helpers and breeders typically live in groups on a single territory, disentangling the effect of helpers on productivity from the underlying effects of the quality of the breeding pair or territory can be very difficult (Brown 1978; Brown et al. 1982; Koenig and Mumme 1990; Magrath and Yezerinac 1997; Cockburn 1998; Dickinson and Hatchwell 2004; Cockburn et al. 2008). In riflemen, adult helpers are often grown offspring from a previous year’s brood, so highly productive pairs are more likely to have kin in the population and hence are more likely to be helped. However, Preston, Briskie, et al. (2013) noted several characteristics of riflemen social organization that make the potential link between breeder/territory quality and the presence of helpers and present productivity less problematic. First, juvenile riflemen always disperse before the next breeding season and will attempt to breed independently before helping. Therefore, successful breeding pairs are not guaranteed to have helpers in later breeding seasons. Second, adult helpers are often failed breeders who have experienced mate loss or nest failure, so to some extent helper availability is driven by such chance events. Third, as in other cooperative breeders with redirected helping (e.g., Dickinson et al. 1996; Nam et al. 2010), breeders may be assisted by nondescendant kin, so the presence of helpers is not conditional on previous success. Finally, territory quality is unlikely to be an important confounding variable because riflemen helpers do not live permanently on the breeder’s territory, instead they have their own territory that they maintain while helping and frequently return to for foraging (Preston SAJ, personal observation). Therefore, we suggest that it is reasonable to conclude that helpers gain indirect benefits from their cooperative behavior.

Of course, close kinship between helpers and recipients does not preclude the possibility of helpers also gaining direct fitness benefits (e.g., Cockburn 1998; Clutton-Brock et al. 2001; Richardson et al. 2002). However, several factors suggest that rifleman helpers do not benefit from the most commonly reported direct fitness gains. First, pairs are sexually monogamous (Preston, Briskie, et al. 2013), excluding the possibility that helpers gain immediate reproductive success through helping. Second, as described above, juveniles disperse before reaching sexual maturity and adult helpers maintain their own home-ranges while helping, in some cases simultaneously caring for their own offspring (Preston, Briskie, et al. 2013). Therefore, hypotheses such “pay-to-stay,” in which helpers benefit by being allowed to live on a high quality territory (Gaston 1978, Mulder and Langmore 1993, Kokko et al. 2002), or benefits associated with group augmentation (Brown 1980, 1987) are unlikely to apply here. Furthermore, the flexible nature of the helping system in riflemen, in which individuals may switch back-and-forth between helping and breeding throughout their lifetime, means that the productivity of a pair is not as tightly linked to the probability of gaining helpers the following year. Therefore, although we cannot rule out the possibility that helpers gain some direct fitness benefit from their cooperative behavior, we think any such benefits are likely to be small. In contrast, our study does provide further evidence that there are substantial indirect benefits associated with helping in the rifleman and that kin selection is likely to have played a significant role in the evolution of cooperative breeding in this species.

## FUNDING

The research was funded by the UK Natural Environment Research Council NE/F009321/1.
